# Eye health in the future: what are the challenges for the next twenty years?

**Published:** 2008-09

**Authors:** Hugh R Taylor

**Affiliations:** Harold Mitchell Professor of Indigenous Eye Health, Melbourne School of Population Health, University of Melbourne, 207 Bouverie Street, Carlton, 3053, Australia.

**Figure:**
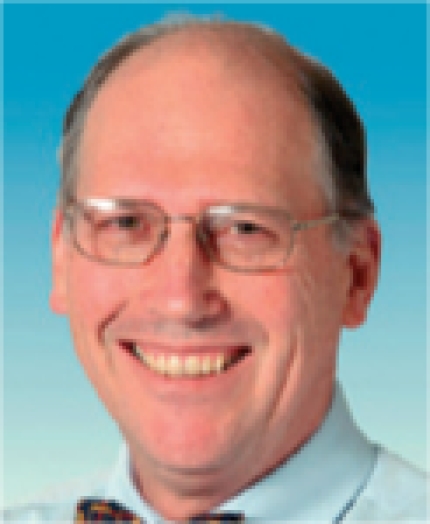


When looking ahead, it is really important to know where we have come from. This allows us to project identified trends and to reflect on the tremendous amount of change that can happen over a relatively short period.

Reflect for a moment on the intensity of the debate regarding the use of intraocular lenses (IOLs) in low-income settings in the 1990s. As they were still very expensive, some insisted that aphakic correction after surgery was the best approach for these countries. The subsequent availability of low-cost IOLs made the debate irrelevant. It dramatically changed our ability to provide modern IOL cataract surgery and control cataract blindness worldwide, and it laid the basis for VISION 2020. Other dramatic changes occurred with the introduction of ivermectin for onchocerciasis and azithromycin for trachoma, which gave us the ability to eliminate these two devastating and previously intractable causes of blindness. Our medical management of glaucoma or of age-related macular degeneration today is totally different from what it was 20 years ago, although there is still a long way to go. Our approach to refractive error has also altered dramatically, thanks to the recognition of its importance and the availability of high-quality, low-cost spectacles.

So, given what we know about the past, what are the challenges we face in the future?

## Doing what we know

### The gap between knowledge and practice

To my mind, the single biggest challenge we face – and the one which will offer by far the biggest pay-off – is the challenge of fully applying what we already know. We know how to cure cataract blindness, how to cure uncorrected refractive error, how to eliminate trachoma and onchocerciasis, and how to prevent most blindness from diabetic retinopathy. We do not need to wait for a new gene to be discovered or a new laser. Why, then, aren't we putting this knowledge into practice right now?

VISION 2020 recognises that three-quarters of all blindness worldwide is either avoidable or preventable with what we currently know and it aims to bridge the existing gap between knowledge and practice. The VISION 2020 initiative emphasises disease control, but it also recognises the need for eye care to be delivered through national programmes that are tailored to individual countries. The initiative also rightly emphasises human resource development and infrastructure.

However, none of this is possible without money! Non-governmental organisations and donor agencies can provide some level of ongoing funding, as well as flexible start-up money for new initiatives. Yet long-term funding for ongoing blindness prevention and eye care must eventually come from governments or their insurance programmes, although individuals themselves will continue to pay some costs.

### The crucial importance of advocacy

What is really needed, therefore, is strong advocacy programmes to alert governments to the importance of eye care, the costs of not doing more, and the benefits of intervening to prevent avoidable blindness.

**Evidence for advocacy.** For effective advocacy, good data is essential.

- National, population-based prevalence data on blindness can be invaluable, but using data from similar countries, extrapolated with national demographic data, is almost as effective and far more cost-effective and timely.
- Exciting advances are being made in quantifying the ‘burden of disease’ or the ‘loss of wellbeing’ attributable to vision loss and translating it into monetary terms (such as US dollar per quality-adjusted life-year or QALY).
- Eye care interventions are amongst the most cost-effective of all health care interventions, in terms of reducing the number of years people would otherwise have lived with a disability (this is quantified as disability-adjusted life-years or DALYs).
- Economic arguments such as these are what governments and finance departments understand. For example, one can show that for each US dollar spent on eye care, there is a US $5 return to the community. Such an objective financial argument carries more weight than an emotional call for action to stop people losing their sight.
- It is also important to monitor and evaluate our programmes to show their ongoing success, efficiency, and impact – and to add this to the case for eye care we are trying to build.

**Figure:**
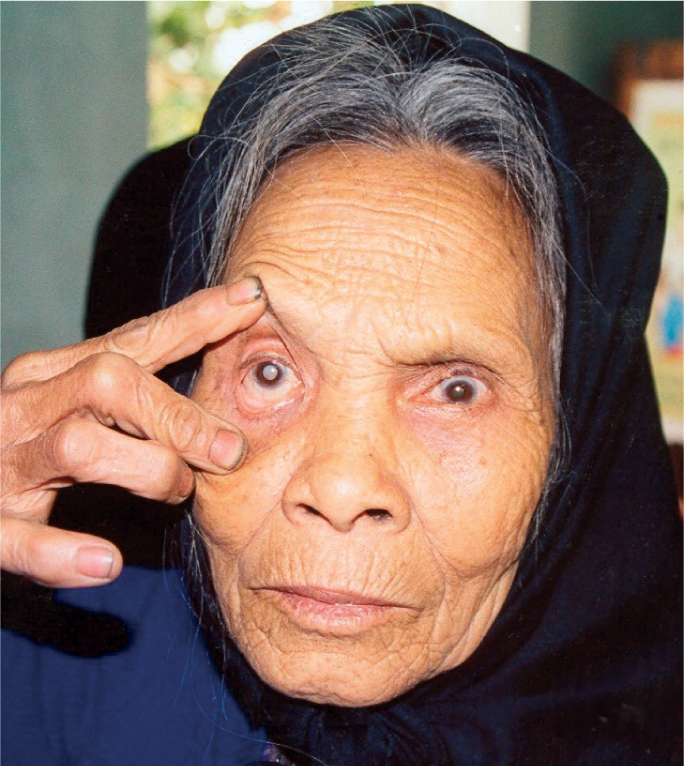
Older woman with cataract. VIET NAM

**Developing advocacy skills.** With solid evidence being available to support the establishment of eye care programmes, the focus moves to the need for strong advocacy skills.

- Key members of eye teams should receive specific training to enable them to succinctly state and present the case for eye health.
- Some work has already started on this – workshops run by the International Agency for the Prevention of Blindness and the International Centre for Eye Health, as well as various activities by the International Council for Ophthalmology – but much more will be needed in the coming years.
- There are a growing number of good examples of successful case development and advocacy at a national level, but these successes will need to be repeated many times, for each country and of course at the international level through the World Health Assembly.

## Recognising the changing demography

### The population is getting older worldwide

- Over the last 50 years, life expectancy has increased by about 20 years around the world, except in those countries most affected by HIV/AIDS. As a consequence, the world population is getting older, with the number of elderly people set to double in the next 20 years.
- Because the frequency of blindness and vision loss increases with age, the demand for eye services will increase exponentially as populations grow older – this will need to be taken into account when planning eye care services. For example, the cataract surgical rate a country will need to achieve in order to eliminate blindness may increase significantly if there are more older people with cataract.
- In addition, the indications for surgery may change over time, as countries grow and people need to be able to read or drive a car in order to remain economically active; this will further increase the need for cataract surgery.

### New patterns of disease

The changing demographic structure, coupled with economic growth, has also led to dramatic changes in the patterns of disease. Diseases particular to older people, especially age-related macular degeneration (AMD) and glaucoma, have become increasingly important.

### AMD and glaucoma

- AMD is now the leading cause of blindness in most developed countries; it was not even listed as an important cause 50 or 100 years ago! Glaucoma too will become increasingly important.
- However, for both AMD and glaucoma, prevention is tough, treatment long and expensive, and at present only partially effective.
- In addition, interventions are very costly, but relatively inefficient, and both diseases require good low vision services to help people make the most of their remaining vision.

### Diabetes

- Vision loss from diabetes is another growing problem resulting from increased life expectancy and changing lifestyles. People who would ordinarily have died of old age now live long enough to experience the retinal complications of the disease (diabetic retinopathy).
- Whereas diabetes once only affected people in high-income countries, the number of people with type-2 diabetes and obesity in low- and middle-income countries is now rapidly growing.
- As ophthalmologists cannot screen every person with diabetes for diabetic retinopathy on an annual basis, there is a pressing need to develop effective screening strategies with teams working in primary care and diabetes clinics. These teams might include ophthalmic nurses, optometrists, or other suitably trained mid-level personnel.

**Changing interventions.** These new patterns of disease will lead to changes in interventions. Ongoing, chronic conditions such as diabetic retinopathy, AMD, and glaucoma require a very different approach from the almost one-off contact required for cataract surgery. For example, persuading diabetes patients to change their behaviour may become an important way to prevent blindness from diabetic retinopathy.

## Therapeutic advances

### AMD treatment

In the field of AMD, I feel sure there will be very exciting and rapid advances, particularly concerning drugs that inhibit or reverse the growth of new vessels; both better compounds and better delivery methods are being developed – specifically, vascular endothelial growth factor (VEGF) blockers or inhibitors (see article on page 50). The challenge will be to make these drugs available at prices both affordable and justifiable on a cost-effectiveness basis. The current drugs are often unaffordable by most (even in high-income countries) and not cost-effective. The biggest pay-off will be for drugs that slow or prevent the progression of the disease.

**Figure:**
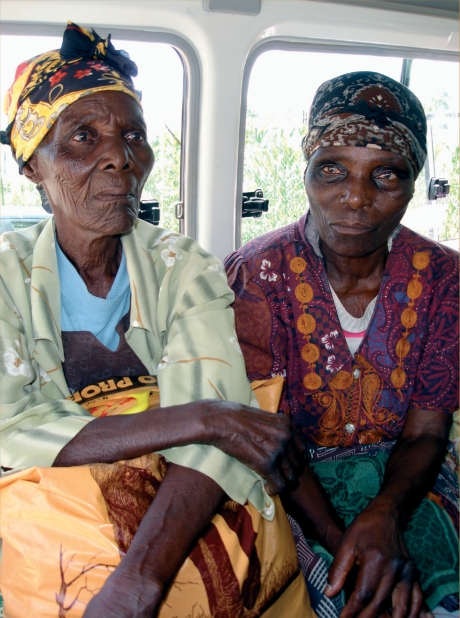
As the frequency of blindness and vision loss increases three times with each decade over 40, the demand for eye services will increase exponentially as the world population grows older

### Glaucoma treatment

In the case of glaucoma treatment, we are also likely to witness the further development of drugs that are more convenient to administer and have fewer side effects. However, these new drugs will have to be far superior to justify prices that are higher than those of the present drugs.

It would be much better if we were able to diagnose the people with glaucoma who are currently undiagnosed (estimated at 50% of all cases). It would also be better if we were able to provide laser trabeculoplasty as the first-line therapy, rather than drugs that are expensive, have more side effects, and are plagued by poor compliance. However, the real breakthrough for glaucoma will be a safe and effective filtering procedure, such as a surgical drainage stent. Advances in our understanding of wound healing and nanofabrication should allow a simple-to-insert stent to give simple surgical control of pressure.

### Gene chips

The ready availability of gene chips in the future could enable improved diagnosis and specifically tailored treatments for various eye diseases, including AMD and glaucoma. Gene chips are already available as prototypes, but there is likely to be much development as more and more genes are identified for various conditions and differential responses to treatment are determined.

### Accommodating IOLs

All of ophthalmology and eye care would be revolutionised by the development of an effective and safe accommodating IOL. This would immediately replace all current IOLs and would further increase the demand for cataract surgery at earlier stages of vision loss. In addition, an accommodating IOL would also replace most refractive surgery and therefore affect both the Excimer laser surgery and the contact lens markets. Moreover, accommodating IOLs could well become the preferred option for the correction of presbyopia and make bifocal spectacles redundant. Wouldn't that be a revolution!

### Bionic eye and stem cell biology

Another exciting possibility – although likely to be out of the financial reach of most people – is the ‘bionic eye’ for those with severe outer retinal damage. Such implants are likely to be extremely expensive and will be far more challenging to develop than the successful bionic ear, which has been in use for 20 years and still costs over US $20,000. Stem cell biology also offers hope for those with conditions which are currently difficult to treat, such as some forms of corneal blindness.

### Childhood blindness

I hope it is only my ignorance, but I do not see any major breakthroughs in childhood blindness. Genetic identification may help to guide planned pregnancies in high-income countries and the first successful examples of gene transfer appear to offer exciting possibilities for treating inherited retinal degeneration. However, neither of these advances is likely to have a major impact in low- and middle-income countries in the foreseeable future. As far as I can see, the major advances will result from doing what we already know how to do, but doing it better: rubella and measles vaccination, clean faces, improving vitamin A intake, improving management of amblyopia (as it is needed after cataract, glaucoma, and squint treatment), monitoring of oxygen levels, early detection and low vision training, etc.

## Conclusion

I will probably be embarrassed by some of these predictions by the time this is published and, surely, many of them will turn out to be foolish with hindsight. However, even if only one or two turn out to be correct, we will have a very exciting time over the next 20 years.

